# Clinical Surgery in the Paris Hospitals

**Published:** 1869-02-15

**Authors:** A. W. Bosworth

**Affiliations:** Paris


### CORRESPONDENCE.

---

### *Clinical Surgery in the Paris Hospitals.*

PARIS, Jan., 1868.

In the service of Mr. Maisonneuve of the Hotel Dieu, saw a man, aged about 50, having an inguinal hernia descending into the scrotum, making it the size of a boy's head. The clever surgeon, immediately on seeing the patient, tapped the tumor slightly with his finger, and, in an instant, made the diagnosis by the sound thus produced. This sound resembled that produced by gently tapping a moistened bladder filled with air. The surgeon's ear once accustomed to it, he has no need of manipulation to make his diagnosis. Calling for a caoutchouc band, three inches wide and about three feet long, he first encircled the neck of the hernia so closely as to prevent the herniary aperture from dilating or bursting; then, with considerable force, enveloped the whole hernia, thus producing a strong pressure. Immediately one could see the tumor forcibly diminish, and within five minutes the greater part of it was reduced. A little gentle taxis completed the operation. This surgeon declares he never saw even a strangulated hernia that he could not reduce with this india-rubber band, which he always employs if he considers the various hernial parts are not too much diseased to contra-indicate reduction.

Although it may seem useless to refer to the long disputed question of a specific cancerous cell, yet, many a practitioner may be pleased to know the opinion expressed this week by so distinguished a man as M. Maisonneuve; a surgeon who probably sees and treats more tumors, called cancers, than all the rest of the surgeons of Paris combined.

Safely can I say that one can never pass through his wards without finding at least a dozen unfortunate beings thus affected. For M. Robin, Professor of Histology, at the Medical Faculty of Paris, the word cancer has no signification, and ought to be obsolete; for him, this so-styled specific cell does not exist, and what has been so considered are only the various phases of morbid development of several different kinds of cells. M. Maisonneuve, at his last lecture, declared that he could not agree with M. Robin when saying there is nothing particular in a cancerous tumor. This surgeon of the Hotel Dieu said, that within fifteen years he had operated on many of these tumors, examined them with the microscope, taken notes of them, which he had put aside. That one day, while reperusing these notes, he had seen that the tumors having a particular cell relapsed within a certain time; and for him they were cancers. That whenever he saw this form  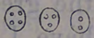 under the microscope, he was sure the patient had not more than five years to live, and that the poor creature would have all the symptoms of a cancerous cachexia.

If he saw this form  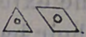 he was quite sure of a final recovery.

If this form,  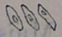 which was formerly considered cancerous, he knew the tumor would not produce the cancerous cachexia.

If he saw the tumor to be an epithelioma, he knew it might relapse at its primitive situation, or in the neighboring ganglions; but, that there would be no cancerous cachexiæ, no cancerous generalization. That, as a general rule, the true cancer usually relapses within three years, and always within five years. That one should operate whenever he saw, by so doing, he might relieve the patient's sufferings for a certain period. In the most of his operations he does not employ the bistoury, but a wedged-like, solid *arrow* as he calls it composed of chloride of zinc and wheat flour, equal parts, water *q. s.*, which he will introduce into any part of the body to a depth proportionate to the tissue affected.

These arrows (flèches) are about three inches long, one-sixth of an inch thick. At the base two-thirds of an inch wide, the apex pointed.

I have seen him remove, by this method, parts of the uterus; often a whole breast; half of the maxillary inferior, parts of the maxillary superior; the bones of the nose, etc. He thrusts them into a part by first making a deep incision merely the width of an ordinary bistoury. The part resting above the surface is cast off, and then the whole is covered with dry lint. About ten days after their insertion an eschar comes away taking all tissues affected for half an inch beyond the surface of the caustic.

By this method he proclaims to avoid, as a general rule, hæmorrhage, erysipelas, and purulent infection. In by far the greater part of his operations such is the case. Often have I seen him successfully operate on poor creatures whom many a skilful surgeon had refused an operation, and would not dare to touch, fearing from the nature of the parts affected, and from the extent of the disease, that the patient would expire in their hands before they could terminate their attempt.

Suffice it to say that although M. Maisonneuve and the Faculty of Paris are not friends, yet I have seen some of the best Professors of the Faculty occasionally employ this method.

For dressing various wounds and operations, Maisonneuve employs the Tincture of Arnica, which he considers to prevent inflammatory action, the Arnica root long having been esteemed as a preventative to purulent infection; glycerine, which Trousseau had long considered beneficial in superficial skin affections, and which Bazin has long praised in the treatment of eczema, zona, ichthyosis and in other skin diseases; French aromatic wine; dry lint, or lint saturated with the different substances here mentioned; carbolic acid, everywhere admitted to prevent fermentation, and consequently, putrefaction, which is a consequence of it. Phenic acid is more often used for dressing than any other substance in his service.

Pieces of linen perforated by many small holes, and covered with a thin layer of cerate, which is seen in so many of the surgical services of Paris, Maisonneuve uses no more. In regard to this latter point I think this clever surgeon has shown sensibility to abandon it; for, let me ask, what good can simple cerate, composed of three parts of sweet almond oil and one part of pure white wax, do, aside from keeping air from a wound? It is well known that simple cerate becomes rancid very promptly, and then has an irritating action. If one chooses to employ medicated cerates, such as the cerate of belladonna used in England, antiseptic, antiherpetic, antisyphilitic cerates, in which the simple or Galian cerate is a mere vehicle, then we see there is a direct object in view. In this letter, Mr. Editor, I intended to have spoken of a new kind of splints for fractures, and of a useful apparel for amputations, employed by M. Maisonneuve, but I find there is no room.

Yours Truly,

A. W. Bosworth.

